# Optimization of NaOH-catalyzed steam pretreatment of empty fruit bunch

**DOI:** 10.1186/1754-6834-6-170

**Published:** 2013-11-29

**Authors:** Won-Il Choi, Ji-Yeon Park, Joon-Pyo Lee, You-Kwan Oh, Yong Chul Park, Jun Seok Kim, Jang Min Park, Chul Ho Kim, Jin-Suk Lee

**Affiliations:** 1 Clean Fuel Department, Korea Institute of Energy Research, Jeongeup, Jeonbuk 580-185, South Korea; 2 Department of Chemical Engineering, Kyonggi University, Jeongeup, Jeonbuk 580-185, South Korea; 3 Applied Microbiology Research Center, Jeonbuk Branch Institute, Korea Research Institute of Bioscience and Biotechnology (KRIBB), Jeongeup, Jeonbuk 580-185, South Korea

**Keywords:** Empty fruit bunch, NaOH-catalyzed, Steam pretreatment, Response surface methodology, Delignification, Optimization, Simultaneous saccharification and fermentation, Ethanol yields

## Abstract

**Background:**

Empty fruit bunch (EFB) has many advantages, including its abundance, the fact that it does not require collection, and its year-round availability as a feedstock for bioethanol production. But before the significant costs incurred in ethanol production from lignocellulosic biomass can be reduced, an efficient sugar fractionation technology has to be developed. To that end, in the present study, an NaOH-catalyzed steam pretreatment process was applied in order to produce ethanol from EFB more efficiently.

**Results:**

The EFB pretreatment conditions were optimized by application of certain pretreatment variables such as, the NaOH concentrations in the soaking step and, in the steam step, the temperature and time. The optimal conditions were determined by response surface methodology (RSM) to be 3% NaOH for soaking and 160°C, 11 min 20 sec for steam pretreatment. Under these conditions, the overall glucan recovery and enzymatic digestibility were both high: the glucan and xylan yields were 93% and 78%, respectively, and the enzymatic digestibility was 88.8% for 72 h using 40 FPU/g glucan. After simultaneous saccharification and fermentation (SSF), the maximum ethanol yield and concentration were 0.88 and 29.4 g/l respectively.

**Conclusions:**

Delignification (>85%) of EFB was an important factor in enzymatic hydrolysis using CTec2. NaOH-catalyzed steam pretreatment, which can remove lignin efficiently and requires only a short reaction time, was proven to be an effective pretreatment technology for EFB. The ethanol yield obtained by SSF, the key parameter determining the economics of ethanol, was 18% (w/w), equivalent to 88% of the theoretical maximum yield, which is a better result than have been reported in the relevant previous studies.

## Background

Amid growing concerns over global warming and oil shortages, the Korean government recently announced an ambitious implementation target for transport biofuels. According to the plan, biofuels will account for about 17% of total transport fuel consumption by 2030 [1]. Since Korea can supply only 30% of the necessary biomass feedstocks, most are imported from foreign countries. Southeast Asia, given its geographical proximity to Korea, is the most promising biomass-supplier region. Its main agricultural crop in this regard is palm oil. Great amounts of palm oil residues, including empty fruit bunch (EFB), fronds, and leaves, are generated annually. Among them, EFB is the most advantageous feedstock for ethanol production, because it does not require collection. Additionally, EFB is obtainable in huge quantities, and can be collected year-round [2].

The key step in the production of bioethanol from EFB is sugar fractionation. If the pretreatment employed can lead to a significant cellulosic and hemicellulosic improvement and is also cost effective, it can be considered to be ideal. Many review papers have reported that pretreatment is one of the most expensive units in the cellulosic ethanol production process and, indeed, that the development of cost-effective pretreatment technologies has become the most important challenge of biorefinement [3-7].

Since most lignocellulosic biomasses have unique physicochemical characteristics, different pretreatment technologies should be applied to maximize sugar recovery during biomass fractionation [8,9]. These pretreatments break the internal lignin and hemicellulose bonds and separate the lignin and hemicellulose fractions that potentially can be converted to useful products. Several investigations have reported very promising results for processes such as alkaline pretreatment [10-12], aqueous ammonia-soaking pretreatment [13], low-acid pretreatment [14], steam pretreatment [2], and sequential pretreatment with diluted acid and then alkali [15]. However, nonetheless, they require further study and testing in order to fulfill specific EFB biorefinement needs [10-15], obtain high glucose yields in enzymatic hydrolysis, and simplify the pretreatment process without ignoring economic concerns.

Steam pretreatment, entailing several minutes’ simultaneous treatment with high-pressure and high-temperature steam, can degrade the complex structure of lignocellulosic biomass. Steam pretreatment of EFB for ethanol production in a palm oil mill is in fact the most economical option implementable. However, the use solely of steam pretreatment might not fully remove lignin, leading to low enzymatic digestibility. Delignified samples, significantly, are more easily hydrolyzed by enzymes than lignin-rich ones. Indeed, lignin is very commonly implicated in the prevention of efficient polysaccharide hydrolysis in the cell walls of lignocellulosic materials. For effective enzymatic hydrolysis, alkaline (NaOH) pretreatment, specifically as a means of reducing the C5 (xylan) and lignin contents in EFB and, thereby, enhancing enzymatic digestibility while remaining within the economic constraints, is necessary. In this work, NaOH-catalyzed steam pretreatment was incorporated into an efficient EFB-pretreatment technology. The effects of several major pretreatment parameters including NaOH concentration, temperature, and reaction time on sugar yields and enzymatic digestibility were investigated, and the pretreatment conditions were optimized. Simultaneous saccharification and fermentation (SSF) subsequently was conducted to determine the fermentability of the pretreated EFB and the ethanol yields.

## Results and discussion

### H_2_SO_4_ and NaOH pretreatment of EFB

For effective fermentation of EFB into bioethanol, pretreatment to reduce its hemicellulose and lignin contents is required. Acid pretreatments have been reported to be effective in removing hemicellulose, as have alkaline pretreatments in removing lignin from biomass, which processes improve the accessibility of cellulose to enzymes. H_2_SO_4_ and NaOH were employed as the acid and alkaline solutions, respectively, in the preliminary pretreatments.

The batch reactions performed in this work are summarized in Table 1. The reactors (internal volume: 13.5 cm^3^)were constructed of 1/2" stainless steel tubing. The reaction temperatures were controlled in oil baths. The initial series of experiments were conducted with 0.2, 0.5, and 0.8 wt.% H_2_SO_4_ and 1.5, 3.0, and 4.5 wt.% NaOH at different temperatures (range: 140 ~ 200°C and reaction times (range: 0 ~ 60 min.). These batch reactions were undertaken in order to compare the characteristics of EFB pretreatment by acid and alkaline solutions. The maximum glucan recovery by these two chemical pretreatments at the optimal reaction time, and the maximum glucose yield by enzymatic hydrolysis of the pretreated EFB, were determined. In the H_2_SO_4_ pretreatment, the glucan recovery was decreased and the glucose yield was increased as the reaction temperature increased. By the batch reaction with 0.8 wt.% H_2_SO_4_ at 200°C for 50 min., the maximum glucan recovery and glucose yield were 63.2% and 73.4%, respectively. By the 3.0 wt.% NaOH pretreatment at 200°C for 40 min., the maximum glucan recovery and glucose yield were 87.4% and 81.4%, respectively. Evidently then, the NaOH pretreatment achieved, under similar reaction conditions, a higher glucan recovery yield than did the H_2_SO_4_ pretreatment. To achieve the maximum glucan recovery and glucose yield in the batch reactions, more than 40 min of reaction time was needed.

**Table 1:** **Maximum glucan recoveries and glucose yields by H**_**2**_**SO**_**4 **_**and NaOH batch reactions**

**Temp. (°C)**	**Concentration of H**_**2**_**SO**_**4 **_**(%)**			**Concentration of NaOH (%)**		
	**0.2**	**0.5**	**0.8**	**1.5**	**3.0**	**4.5**
140	95.5^**a**^ / 53.1^**b**^	94.2 / 54.3	93.0 / 55.6	98.1 / 50.4	97.3 / 51.6	97.0 / 50.0
160	90.1 / 58.8	88.6 / 60.3	85.3 / 61.7	94.5 / 56.6	94.3 / 58.7	94.2 / 61.6
180	77.4 / 62.8	75.8 / 63.1	71.6 / 63.9	89.3 / 67.3	89.1 / 71.5	87.6 / 73.2
200	69.9 / 69.4	68.0 / 72.6	63.2 / 73.4	88.0 / 78.1	87.4 / 81.4	87.1 / 78.5

**a**: maximum glucan recovery after batch pretreatment.

**b**: maximum glucose yield by enzymatic hydrolysis with pretreated biomass.

A large-scale (1.0 L) batch reactor was used so as to maximize the cellulose recovery from EFB by H_2_SO_4_ treatment at reaction temperatures between 121 and 190°C. Reaction times and H_2_SO_4_ concentrations in the 1.06 ~ 240 min and 0.2 ~ 0.8% ranges, respectively, were tested. The reaction temperatures were controlled by insertion of live steam and operation of an electrical heating jacket covering the outside of the reactor. In the steam pretreatment, a biomass was simultaneously treated with high-pressure and high-temperature steam for several minutes in order to degrade the complex lignocellulosic structure, This successfully reduced the reaction time to the pretreatment optimum. Under these runs with a similar Severity Index (2.7-3.1) [16], C6 (glucan) was preserved at a higher than 88.6% level in the pretreated biomass basis of raw EFB, while 80% of C5 (xylan) was released to the pretreated liquor. The delignification relative to the raw EFB, however, was lower than 35%. In these runs, the enzymatic digestibility of the pretreated biomass obtained with a 30 FPU dosage of enzyme was lower than 50%, insufficient for ethanol conversion. That lignin is a factor hindering the enzymatic hydrolysis of lignocellulose is well-documented [17,18].

With NaOH treatment, the processes were conducted at reaction temperatures between 120 and 170°C, over reaction times between 4.2 min and 120 min, and with an NaOH concentration of 1.0%. The high reaction temperatures coupled with relatively longer reaction times induced severe degradation of the solubilized carbohydrates (C5, C6) along with higher delignification.

The enzymatic digestibility tests carried out using pretreated EFB contained relatively higher and lower amounts of C6 (glucan) and lignin, relatively. Under the NaOH pretreatment conditions, that is, a reaction time of 4 min 32 sec and a temperature of 170°C, the glucan yield was 92% that of the raw EFB, and the enzymatic digestibility of the pretreated EFB was 87%.

In comparing the H_2_SO_4_ and NaOH pretreatments of EFB, the glucan recoveries were found to be similar. The H_2_SO_4_ pretreatment was more effective for C5 hydrolysis than delignification; the NaOH pretreatment achieved higher delignification, and obtained the same profile of enzymatic digestibility. In both cases, the rate and extent of the enzymatic hydrolysis of the biomass correlated better with the removal of alkaline-insoluble lignin than with the removal of xylan [19].

### NaOH-catalyzed steam pretreatment

The alkaline impregnation (NaOH-soaking) stage was introduced to improve the enzymatic digestibility of EFB. Complementarily, NaOH-catalyzed steam pretreatment was conducted for enhanced EFB-pretreatment effectiveness. The fractionation process variables, including reaction temperature, reaction time, and NaOH concentration, were selected, by preliminary tests, as the experimental design (response surface methodology: RSM) factors. The EFB was pre-soaked in 0.5 ~ 5.5% concentrations of NaOH at room temperature for 12 h by application of pressurized steam at temperature of 127 ~ 193°C for times ranging from 4 min 40 sec to 11 min 20 sec using a 1.0 L batch reactor. The initial biomass loading was 60 g, and solid/liquid ratio during pretreatment was 1/3 ~ 1/8. The compositional changes in the solid samples (*p* ≤ 0.05), the solid remaining, and the contents of glucan and xylan after pretreatment varied according to the reaction temperature and NaOH concentration (Table 2). From the RSM analysis, the following solid remaining, glucan and xylan recovery and enzymatic digestibility values were obtained:

$$\begin{array}{l} A=159.213-0.792x+1.932y+0.003\mathrm{xx}+0.308\mathrm{yy}\\ \;+0.220\mathrm{zz}-0.041\mathrm{xy}-1.416\mathrm{yz}-0.029\mathrm{zx}\\ \;+0.008\mathrm{xyz}\;\left(R^{2}=0.888\right) \end{array}$$

$$\begin{array}{l} B=172.351-0.659x-4.790y+0.003\mathrm{xx}\\ \;+0.552\mathrm{yy}-0.708\mathrm{zz}-0.034\mathrm{xy}-0.809\mathrm{yz}\\ \;+0.002\mathrm{zx}+0.007\mathrm{xyz}\;\left(R^{2}=0.523\right) \end{array}$$

$$\begin{array}{l} C=211.006-1.063x-2.151y\\ \;+0.003\mathrm{xx}+0.425\mathrm{yy}-1.142\mathrm{zz}-0.039\mathrm{xy}-0.849\mathrm{yz}-0.012\mathrm{zx}\\ \;+0.009\mathrm{xyz}\;\left(R^{2}=0.731\right) \end{array}$$

$$\begin{array}{l} D=-88.473+1.759x-1.842y-0.006\mathrm{xx}-0.118\mathrm{yy}\\ \;-2.552\mathrm{zz}+0.020\mathrm{xy}+1.736\mathrm{yz}+0.125\mathrm{zx}\\ \;-0.007\mathrm{xyz}\;\left(R^{2}=0.964\right) \end{array}$$

where A is the solid remaining, B is the C6 recovery, C is the C5 recovery, D is the enzymatic digestibility, x is the temperature, y is the time, and z is the NaOH concentration.

**Table 2:** NaOH-catalyzed steam pretreatment conditions, composition of pretreated biomass, and enzymatic digestibility

**No.**	**Temp. (°C)**	**Time (min)**	**Concentration of NaOH soaked (%)**	**Solid remaining (%)**	**Waste liquor (ml)**	**Composition of pretreated EFB (%)**			**Recovery (%)**		**Enzymatic digestibility (%)**
						**C6 (glucan)**	**C5 (xylan)**	**Lignin**	**C6 (glucan)**	**C5 (xylan)**	
1	127	8	3.0	73.5	175	46.5	29.1	18.4	87.0	83.3	77.7
2	140	6	3.0	74.3	255	48.6	29.7	19.0	92.0	85.8	73.1
3	140	8	1.5	81.3	150	44.9	28.3	19.8	93.0	89.6	63.4
4	140	10	4.5	66.8	244	52.6	29.5	17.0	89.5	76.6	86.5
5	160	6	4.5	63.8	360	56.0	28.8	14.6	91.0	71.5	89.7
6	160	10	1.5	77.3	350	45.0	27.0	21.4	88.6	81.1	61.7
7	160	4 min 40 sec	3.0	70.7	335	53.1	27.5	16.1	95.5	75.5	80.1
8	160	8	0.5	80.0	255	43.9	25.0	22.1	89.5	77.9	46.3
9	160	11 min 20 sec	3.0	68.0	430	53.7	29.4	14.3	93.1	77.7	88.8
10	160	8	5.5	54.5	410	56.4	25.4	15.8	77.8	51.6	93.5
11	160	8	3.0	60.1	365	52.7	26.7	15.7	80.2	63.5	83.8
12	160	8	3.0	70.2	375	50.1	28.2	16.6	89.5	76.9	87.8
13	160	8	3.0	69.3	400	54.5	30.1	15.2	96.2	81.1	84.1
14	180	6	1.5	74.0	504	49.9	28.7	18.8	94.1	82.5	62.2
15	180	8	4.5	61.2	446	58.7	28.8	13.3	91.4	68.6	89.6
16	180	10	3.0	65.7	515	56.4	29.9	14.0	94.4	76.5	87.3
17	193	8	3.0	63.8	480	58.0	27.0	13.8	94.3	67.0	80.7

**Figure 1:**
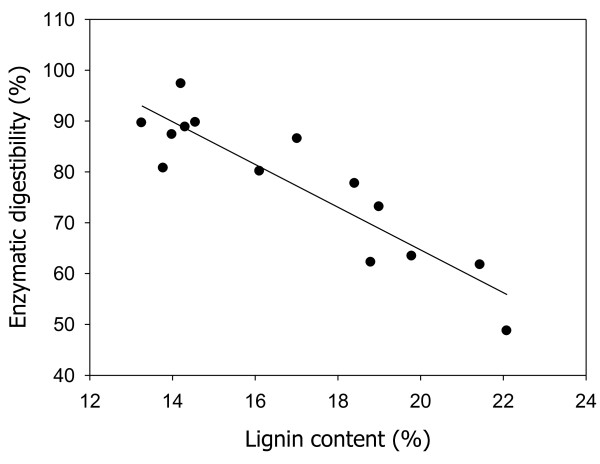
Effects of lignin contents on enzymatic digestibility in pretreated EFBs.

As can be observed, there was little effect by the cellulosic composition change on the reaction temperature or reaction time, suggesting that after the NaOH-catalyzed steam treatment, the C6 (glucan) and C5 (xylan), due to their rigid structures, remained largely intact in the biomass. By contrast, the lignin levels were substantially affected by the concentration of NaOH. In fact, in this study, NaOH was the most effective variable with respect to the delignification of EFB. Under the same pretreatment conditions, the higher-concentration NaOH-catalyzed steam treatment removed more lignin from the EFB. Moreover, the C6 (glucan) and C5 (xylan) contents in the pretreated samples were increased (Table 2). The C6 (glucan) recovery was higher than 80% in the solid fraction under all of the above-noted reaction conditions, and approximately half of the C5 (xylan) was solubilized even at high NaOH dosages. Additionally, the enzymatic digestibility of the pretreated samples was increased. The most significant such increase was a function of the lignin content of a pretreated sample (Figure 1). This indicated that the lignin content had the greatest impact on biomass digestibility, due to the enzymes adsorbed onto making the enzyme ineffective, and, further, that the lignin played a larger role than the xylan as an enzyme-reaction resister. Delignification was particularly important, in that the CTec2, the cellulase enzymes applied for enzymatic hydrolysis, seemed to have a higher affinity for lignin than do other commercial cellulase enzyme products [20,21]. A clear correlation was observed between the enzymatic digestibility and the lignin content of the pretreated EFB. The enzymatic digestibilities of the pretreated EFBs, which had been more than 85% delignified (lignin content: < 15%) relative to the raw EFB, were higher than 85%. In the case of the pretreated EFBs containing a higher than 20% lignin proportion, the enzymatic digestibility was decreased, dramatically, below 60%. According to these findings, 80 ~ 85% of lignin should be removed from EFB in order to hydrolyze more than 80% of cellulose by CTec2.

The carbohydrate (glucan and xylan) level was directly and positively related to the yields of fermentable sugars and ethanol; therefore, minimization of carbohydrate loss during the pretreatment process is important [8]. Moreover, when the SSF of the treated solids was considered, the amount of residual carbohydrates was, again, directly related to the ethanol yield. Correspondingly, optimal pretreatment conditions must be determined based not only on higher delignification but also on higher carbohydrate retention.

For the purposes of maximizing C6 (glucan) and C5 (xylan) recovery as well as enzymatic digestibility, the optimal conditions of NaOH-catalyzed steam pretreatment were determined. The particulars were as follows: 3% NaOH impregnation (soaking during 12 h under an ambient room temperature) by 160°C steam treatment for 11 min 20 sec of reaction time. Under these conditions, the recoveries of glucan and xylan were 92% and 78%, respectively, and the enzymatic digestibility was 87% using an enzyme dosage of 30 FPU/g glucan (Table 2). The carbohydrates (glucan and xylan) of EFB were thus well-preserved in the solid fraction during the NaOH-catalyzed steam pretreatment with high delignification of the liquid fraction, which is a very important beneficial factor in bioethanol production overall.

### SSF of pretreated EFB

**Figure 2:**
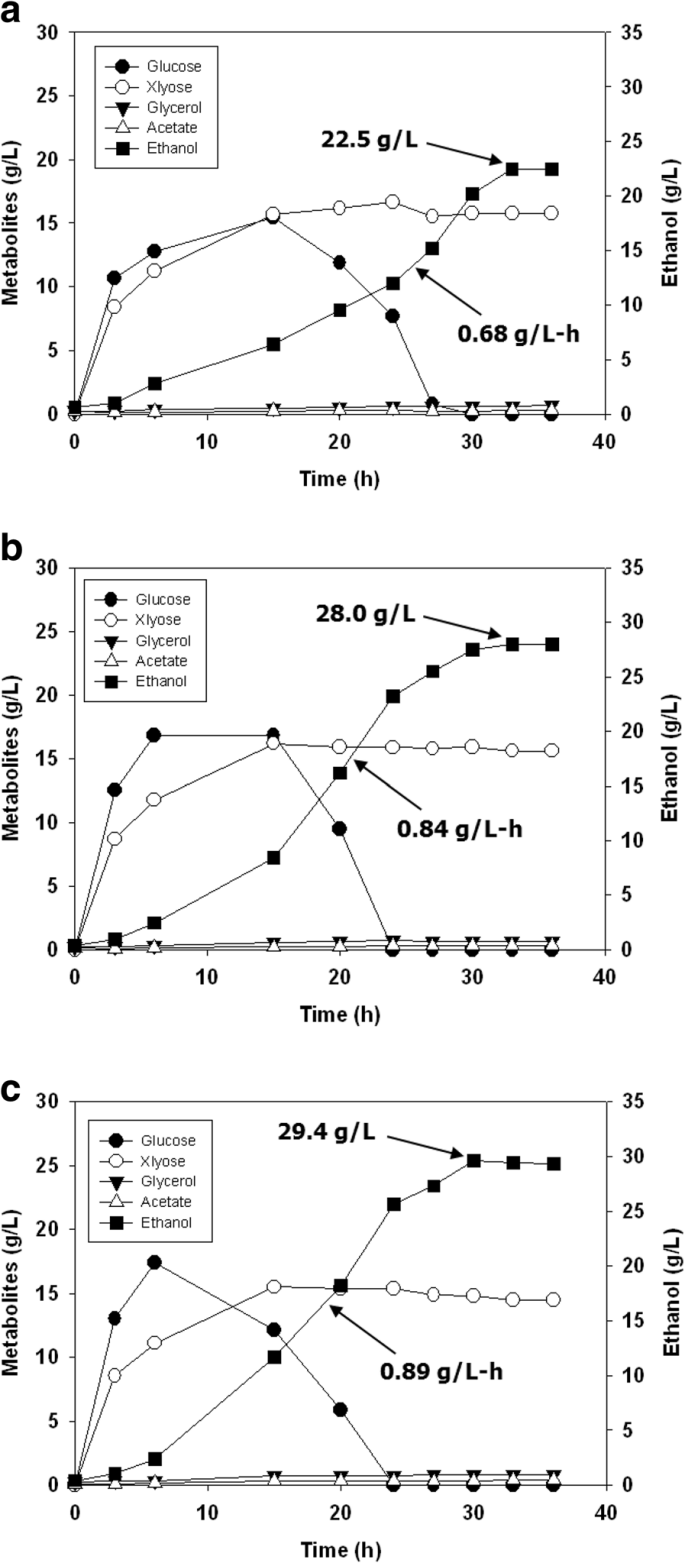
**Time course of SSF of NaOH-soaked EFB (10% w/w) pretreated under different conditions: (a) 1.5% NaOH, 160°C, 10 min; (b) 3.0% NaOH, 160°C, 10 min; (c) 3.0% NaOH, 160°C, 11 min 20 sec.** The SSF was performed with 40 FPU of Cellic CTec2/g glucan at 30°C and 200 rpm for 36 h in a 500 ml flask.

The efficiencies of the pretreatment methods were evaluated by SSF. When the three kinds of pretreated EFB solids (1.5% NaOH for 10 min, 3% NaOH for 8 min, and 3% NaOH for 11 min 20 sec) were applied at a concentration of 10% (w/w) with cellulase (40 FPU/glucan), almost all of the glucans were saccharified, thereby affording an ethanol yield that was more than 88% of the theoretical yield. The highest ethanol yield was, as correspondent with the results of enzymatic saccharification, obtained with EFB solids pretreated with 3% NaOH for 11 min 20 sec. And as expected, a higher saccharification yield was obtained in the SSF process than in the saccharification reaction, due to the release of product inhibition on the enzymatic activity by consecutive conversion of generated glucose to ethanol. Under the pretreatment and SSF conditions, the ethanol yields from the EFB lignocellulosic materials were estimated to be 80.2%, 85.2%, and 88.0% of the theoretical yield, respectively (Figure 2, Table 3).

**Table 3:** Summary of SSF conditions

**Pretreatment conditions**	**Cellulase loading (FPU/g glucan)**	**Maximum ethanol concentration (g/L)**	**Productivity (g/L-h)**	**Ethanol yield, %**^**3**^
1.5% NaOH, 160°C, 10 min^1^	40	22.5	0.68	80
3% NaOH, 160°C, 8 min^1^		28.0	0.84	85
3% NaOH, 160°C, 11 min 20 sec^1^		29.4	0.89	88
3% NaOH, 160°C, 11 min 20 sec^1^	20	23.7	0.72	71
	40	25.3	0.77	76
	60	29.4	0.89	88
3% NaOH, 150°C, 30 min^2^	40	25.6	0.78	78

1: SSF in 500 ml flask.

2: SSF in 5 L fermentor.

3: Based on the theoretical yield: 0.51 g/g.

**Figure 3:**
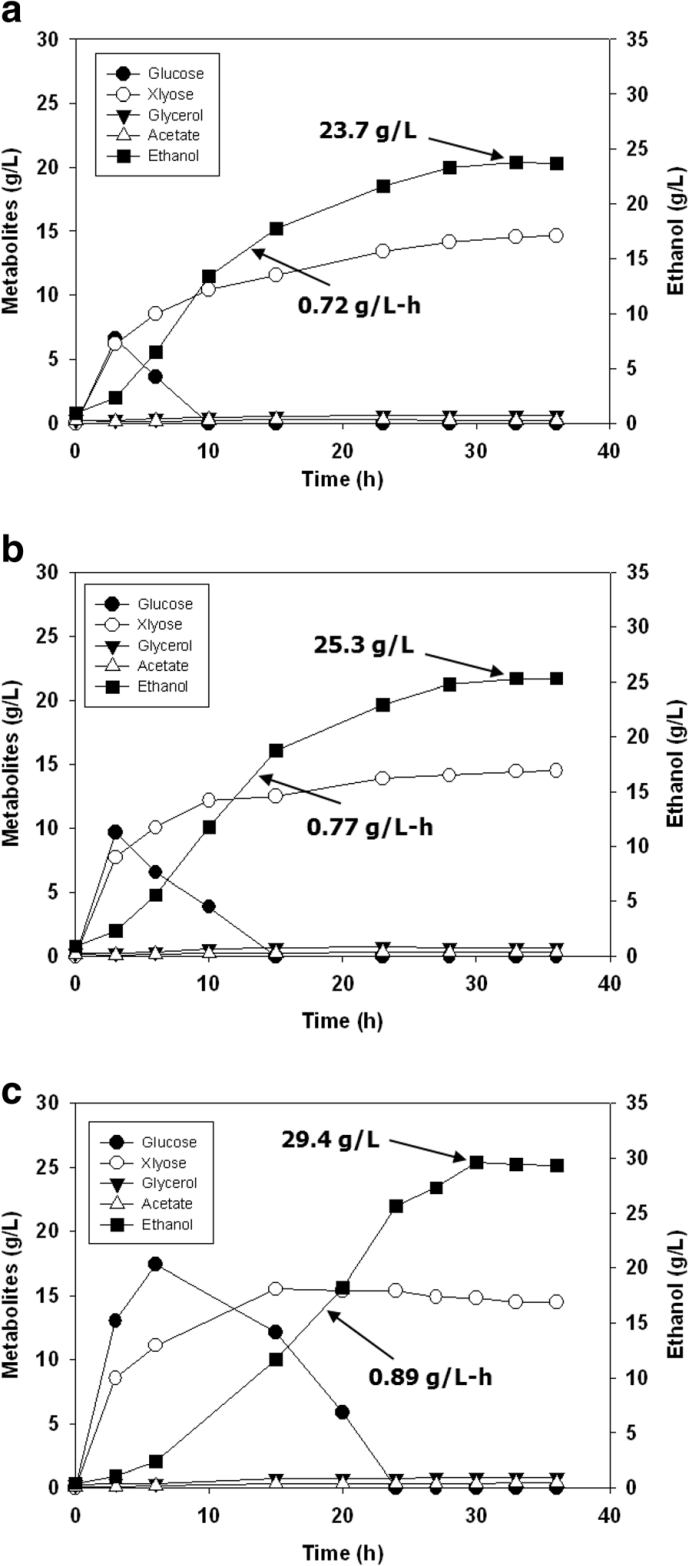
**Time course of SSF of 3% NaOH-soaked EFB (10% w/w) pretreated under 160°C, 11 min 20 sec condition with different Cellic CTec2 cellulase loadings: (a) 20 FPU/g glucan; (b) 40 FPU/g glucan; (c) 60 FPU/g glucan.** The fermentation was performed at 30°C and 200 rpm for 36 h in a 500 ml flask.

**Figure 4:**
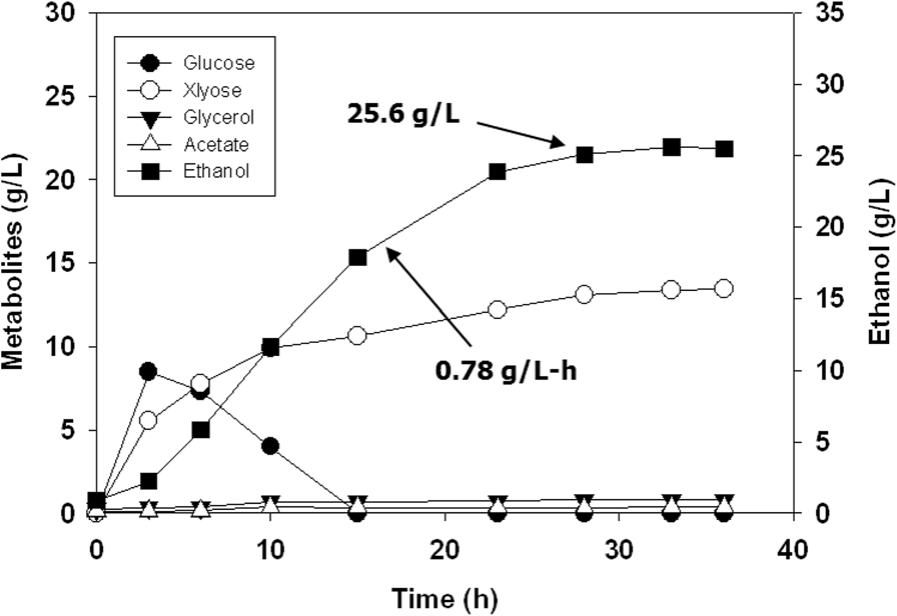
**Time course of SSF of 3% NaOH-soaked EFB (10% w/w) pretreated under 150°C, 30 min condition with 40 FPU/g glucan.** The fermentation was performed at 30°C and 200 rpm for 36 h in a 5 L fermentor.

Subsequently, using the pretreated EFB that had provided the highest ethanol yield, the effects of enzyme loadings on the SSF performances were evaluated. When the enzyme amounts were reduced from 40 FPU/g to 30 FPU/g and 20 FPU/g, the ethanol yields were proportionally decreased (Figure 3, Table 3). Next, an SSF experiment using a 5 L bioreactor was conducted under the same conditions as those holding for the flask experiments (Figure 4). In the results, the ethanol yield was slightly decreased, to 78% of the theoretical yield. Thus, the reproducibility of SSF scale-up from 50 ml in 500 ml flasks to 500 ml in a 5 L bioreactor was determined to be fairly good.

### Overall mass balance

**Figure 5:**
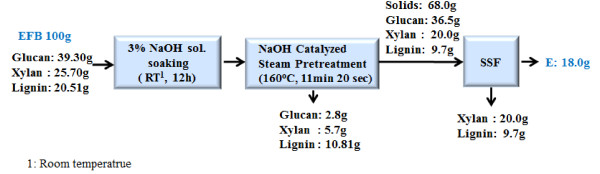
Mass balance for NaOH-catalyzed steam pretreatment of EFB (3% NaOH soaked, 160°C, 11 min 20 sec).

Pretreatment of EFB by the NaOH-catalyzed steam process was highly effective in reducing the lignin content and enhancing the enzymatic digestibility. The ethanol SSF of the pretreated EFB using NaOH-catalyzed steam was conducted without any disturbance. The process, achieving an overall mass balance of raw EFB by NaOH-catalyzed steam pretreatment complemented by SSF, is summarized in Figure 5. For the pretreatment stage, 68.0 g of solid residues were obtained from a solid fraction based on an initial 100 g of dry EFB. The rest of it was released from the liquid fraction as minor contents of glucan and xylan and major contents of lignin including degraded materials. It may be assumed that the removal of lignin by NaOH-catalyzed steam pretreatment greatly increased the porous surface area of the biomass, which in turn improved the accessibility of the pretreated EFB to cellulase enzymes. The pretreated EFB (68.0 g) contained the carbohydrates in the forms of 36 g of glucan and 20 g of xylan, which were fermentable sugars and ethanol to be converted ideally. However, in our enzyme system, the glucan could be a countable sugar, and glucose was the available carbon source for ethanol fermentation. After the SSF of the pretreated EFB was conducted, 18.0 g of ethanol was, finally, obtained. This was the overall ethanol yield by NaOH-catalyzed steam pretreatment of EFB, the most promising EFB-pretreatment method derived to date (Table 4).

**Table 4:** Comparison of EFB-pretreatment performances

**Pretreatment technology**	**Glucan yields (%)**	**Enzymatic digestibility (%)**	**Ethanol yields (g/ 100 g EFB)**	**References**
3% NaOH-soaked - steam pretreatment	92	87 (30 FPU)	18.0	This work
Aqueous ammonia	78.3	41.4	7.3	13
2% NaOH - 121°C	60	70	5.2	11
Steam	89	30	5.0	2
4% NaOH	92	86.4	13.6	10
H_2_SO_4_	82.1	88.6 (60 FPU)	17.5	14

## Conclusions

In the overall results, EFB delignification was particularly important to hydrolysis by CTec2. The level of EFB delignification necessary for effective enzymatic digestibility was 85%. The NaOH-catalyzed steam pretreatment of EFB was highly effective in removing lignin, enhancing enzymatic hydrolysis, and maximizing ethanol yield. This process integrated the advantages of the effects of alkaline (NaOH) soaking and steam pretreatment under a short reaction time. The determined optimal conditions were 3% NaOH impregnation (soaking for 12 h under ambient room temperature) and 11 min 20 sec steam-treatment reaction time at 160°C. The final ethanol yield was 0.18 g/g EFB. This study showed that delignification of EFB coupled with SSF can enable the obtainment of high ethanol yields from biomass.

## Methods

### Raw materials

EFB was obtained from a local palm oil mill in Saba, Malaysia. The EFB was dried in an oven at 45°C for 48 h, milled, screened to select the less-than-5 mm particle size fraction, and homogenized in a single set. The chemical composition of the representative feedstock was as follows: 36.3% glucan, 21.4% xylan, 21.3% Klason lignin. It was analyzed following the standard methods for determination of sugar, lignin, and ash contents [22].

Additionally, for the purposes of enzymatic digestibility tests, Cellic® CTec2 cellulase was purchased from Novozymes Korea (Seoul, Republic of Korea) and used for enzymatic digestibility tests.

### Pretreatment, enzymatic hydrolysis, and SSF

Sixty grams of dried EFB was soaked in 300 ml of a certain concentration of sodium hydroxide solution at room temperature. The slurry was allowed to stand overnight and then was filtered (Whatman No. 1 glass filter) to recover the insoluble solids. The recovered solids were washed with distilled water several times and transferred into an autoclave (working volume: 1 L) preparatory to steam pretreatments. The steam pretreatments were conducted under the designated temperature and reaction-time conditions in a vessel pressurized by nitrogen to 20 bar.

The enzymatic digestibility test was performed in duplicate according to the NREL standard procedures [23]. The enzymatic digestibility was defined as the percentage of theoretical glucose remaining after 72 h of incubation with cellulase enzyme. The enzyme loading was 40 FPU of CTec2/g-glucan of biomass supplemented without β-glucosidase. The enzymatic digestibility test conditions were 50°C and pH 4.8 (50 mM sodium citrate buffer) in a shaker bath agitated at 200 rpm.

To investigate the fermentability of the pretreated EFB, SSF was performed. *Saccharomyces cerevisiae* L3262a obtained from the Korean Collection for Type Culture (KCTC, Daejeon, Republic of Korea) was used in the SSF. Seed cultures were prepared in a 100 mL YPD medium in a 500-mL Erlenmeyer flask, and were grown at 30°C in a shaking incubator at 200 rpm for 12 h until the OD at 600 nm was 10, indicating a dry cell weight of 0.5 g. The SSF was performed using 10% (w/w)-pretreated EFB as a substrate at a 50 ml culture volume in a 500 mL flask with certain cellulase loadings specified in the text. The seed culture, prepared previously, was inoculated into a fermentation medium at a concentration of 5% (v/v). The cultures were grown as they were agitated at 200 rpm. SSF was performed also in a 1 L working-volume medium in a 5 L stirring bioreactor (Bioengineering, Switzerland). The pH of each culture was adjusted to 5.2 ± 0.2 with sulfuric acid, and cultures were grown as they were agitated at 200 rpm. Fermentation samples were clarified by centrifugation for 10 min at 10,000 × g, filtered with 0.2-μm filters and injected into the analytical HPLC system.

### Analysis methods

The concentrations of sugars in the acid hydrolysate were analyzed by high-performance liquid chromatography (HPLC, Waters, USA). The HPLC system consisted of a 1525 HPLC pump, a 717 plus autosampler, a 2487 UV absorbance detector, and a 410 refractometer. The analytical column used was an Aminex HPX-87H column for sugar and organic-acid analysis (Bio-Rad Laboratories, Richmond, CA). The operating temperature of the column was maintained at 85°C. The mobile phase was a 5 mM H_2_SO_4_ aqueous solution to which a volumetric flow rate of 0.6 ml/min was applied. The sample-injection volume was 20 μl.

The concentrations of sugars and metabolites in the fermentation samples were analyzed by high-performance liquid chromatography (HPLC) with a YL 9170 refractive index detector (Young-Lin, Republic of Korea) and a Rezex ROA-Organic Acid H + column 7.8 × 300 mm (Phenomenex, USA) at 65°C. The mobile phase was 2.5 mM sulfuric acid at a flow rate of 0.5 mL/min. The ethanol yield was calculated as the percentage of the theoretical ethanol yield of 0.51 g ethanol per gram of glucose.

## Abbreviations

EFB: Empty fruit bunch; SSF: Simultaneous saccharification and fermentation; C6: Glucan; C5: Xylan; RSM: Response surface methodology; IU: International unit; HPLC: High-pressure liquid chromatography.

## Competing interests

The authors declare that they have no competing interests.

## Authors’ contributions

WIC prepared the study plan and performed the steam pretreatments, while JYP, JPL, and YKO did the work on alkali pretreatment, enzymatic hydrolysis, and compositional analysis. JMP and CHK dealt with the SSF cases and the setup of the mass balance. JSK and JSL coordinated the overall study and prepared the full manuscript. All of the authors read and approved the final manuscript.

## References

[B1] Lee JS Lee JP Park JY Lee JH Park SC Status and perspectives on bioenergy in Korea Renew & Sustain Energy Rev 2011 15 4884 4890 10.1016/j.rser.2011.07.065

[B2] Shamsudin S Shah UK Zainudin HI Aziz SA Kamal SMM Shirai Y Hassan MA Effect of steam pretreatment on oil palm empty fruit bunch for the production of sugars Biomass Bioenergy 2012 36 280 288

[B3] Alvira P Tomas-Pejo E Ballesteros M Negro MJ Pretreatment technologies for an efficient bioethanol production process based on enzymatic hydrolysis Bioresour Technol 2009 101 13 4851 4861 20042329 10.1016/j.biortech.2009.11.093

[B4] Agbor VB Cicek N Sparling R Berlin A Levin DB Biomass pretreatment: fundamentals toward application Biotechnol Adv 2011 29 675 685 10.1016/j.biotechadv.2011.05.005 21624451

[B5] Hendricks ATWM Zeeman G Pretreatments to enhance the digestibility of lignocellulosic biomass Bioresour Technol 2008 100 10 18 18599291 10.1016/j.biortech.2008.05.027

[B6] Hayes DJM Second-generation biofuels: why they are taking so long Energy Environ 2013 2 304 334

[B7] Njoku SI Uellendahl AH Pretreatment as the crucial step for a cellulosic ethanol biorefinery: testing the efficiency of wet explosion on different types of biomass Bioresour Technol 2012 124 105 110 22989639 10.1016/j.biortech.2012.08.030

[B8] Lee JS Parameswaran B Lee JP Park SC Recent developments of key technologies on cellulosic ethanol production JSIR 2008 67 865 873

[B9] Timilsena YP Abeywickrama CJ Rakshit SK Brosse N Effect of different pretreatments on delignification pattern and enzymatic hydrolysability of miscanthus, oil palm biomass and typha grass Bioresour Technol 2013 135 82 88 23069607 10.1016/j.biortech.2012.09.010

[B10] Han M Kim Y Kim SW Choi GW High efficiency bioethanol production from OPEFB using pilot pretreatment reactor J Chem Technol Biotechnol 2011 86 1527 1534 10.1002/jctb.2668

[B11] Piarpuzan D Quintero JA Cardona CA Empty fruit bunches from oil palm as a potential raw material for fuel ethanol production Biomass Bioenergy 2011 35 1130 1137 10.1016/j.biombioe.2010.11.038

[B12] Tan L Yu Y Li X Zhao J Qu Y Choo YM Loh SK Pretreatment of empty fruit bunch from oil palm for fuel ethanol production and proposed biorefinery process Bioresour Technol 2013 135 275 282 23186670 10.1016/j.biortech.2012.10.134

[B13] Jung YH Kim IJ Han JI Choi IG Kim KH Aqueous ammonia pretreatment of oil palm empty fruit bunches for ethanol production Bioresour Technol 2011 102 20 9806 9809 10.1016/j.biortech.2011.07.050 21852123

[B14] Jung YH Kim IJ Kim HK Kim KH Dilute acid pretreatment of lignocellulose for whole slurry ethanol fermentation Bioresour Technol 2013 132 109 114 23395763 10.1016/j.biortech.2012.12.151

[B15] Kim S Park JM Seo JW Kim CH Sequential acid-/alkali-pretreatment of empty palm fruit bunch fiber Bioresour Technol 2012 109 229 233 22306078 10.1016/j.biortech.2012.01.036

[B16] Overend RP Chornet E Fractionation of lignocellulosicsby steam-aqueous pretreatments Philos Trans R Soc Lond 1987 321 523 536 10.1098/rsta.1987.0029

[B17] Mooney CA Mansfield SD Touhy MG Saddler JN The effect of initial pore volume and lignin content on the enzymatic hydrolysis of softwood Bioresour Technol 1988 64 113 119

[B18] Schwald W Brownell HH Saddler JN Enzymatic hydrolysis of steam treated aspen wood: Influence of partial hemicellulose and lignin removal prior to pretreatment J Wood Chem Technol 1988 8 4 543 560 10.1080/02773818808070700

[B19] Kim TH Kim JS Changshin S Lee YY Pretreatement of corn stover by aqueous ammonia Bioresour Technol 2003 90 39 47 10.1016/S0960-8524(03)00097-X 12835055

[B20] Siqueria G Varnai A Ferraz A Milagres MF Enhancement of cellulose hydrolysis in sugarcane bagasse by the selective removal of lignin with sodium chlorite Appl Energy 2013 102 399 402

[B21] Huang R Su R Qi W He Z Understanding the key factors for enzymatic conversion of pretreated lignocellulose by partial least square analysis Biotech Prog 2010 26 2 384 391 10.1002/btpr.324 19938060

[B22] Sluiter A Hames B Ruiz R Scarlata C Sluiter J Templeton D Determination of structural carbohydrates and lignin in biomass 2011 Golden CO: National Renewable Energy Laboratory Report No. TP-510-42618

[B23] Selig M Weiss N Ji Y Enzymatic saccharification of lignocellulosic biomass 2008 Golden CO: National Renewable Energy Laboratory Report No. TP-510-42629

